# Introduction and utility of liquid-based cytology on aspiration biopsy of peripheral nodular lesions of the lung

**DOI:** 10.3892/ol.2013.1763

**Published:** 2013-12-17

**Authors:** JOHJI IMURA, KAORI ABE, YOSHIAKI UCHIDA, MASAHARU SHIBATA, KAZUE TSUNEMATSU, MOTOHIRO SATHOH, SHIGEHARU MIWA, TAKAHIKO NAKAJIMA, KAZUHIRO NOMOTO, SHINICHI HAYASHI, KOICHI TSUNEYAMA

**Affiliations:** 1 Department of Diagnostic Pathology, Graduate School of Medicine and Pharmaceutical Sciences, University of Toyama, Toyama 930-0194, Japan; 2 Department of Pathology, Ibaraki Prefectural Central Hospital, Kasama 309-1793, Japan; 3 Department of Radiology, Ibaraki Prefectural Central Hospital, Kasama 309-1793, Japan

**Keywords:** liquid-based cytology, computed tomography-guided aspiration biopsy/cytology, lung tumor

## Abstract

In the present study, aspiration biopsy cytology (ABC) was used for the diagnosis of peripheral nodular lesions in the lung (PNLL), and liquid-based cytology (LBC) was carried out on the material collected to evaluate it in comparison with the conventional method (CM). The subjects comprised 130 cases that underwent computed tomography (CT)-guided ABC for PNLL. A total of 73 cases received a tumor resection, with a diagnosis based on the pathology, while 57 cases were followed up, as the tumor showed no change on the radiological examinations. Biopsy samples from these patients and lavage fluid from the aspiration needles were used for analysis. Cellular material was obtained by centrifugation of the lavage fluid, and samples were prepared by two methods, direct smearing and LBC according to the ThinPrep method. The samples were categorized into three diagnoses: i) Benign, ii) suspicion of malignancy and iii) malignant. Appropriate samples were collected in 72% of cases by LBC, but only in 36% of cases by the CM. There was no marked difference in cellular images between the two methods, with the exception of a few specific cases. LBC on its own provided sensitivity at 68%, specificity at 61% and accuracy at 65%, while a combination of LBC and biopsy markedly improved these figures to 94, 81 and 84%, respectively. The introduction of LBC is considered useful for the cytopathological diagnosis of PNLL by CT-guided ABC. LBC enables the examination of appropriate samples rich in cellular components and supports a biopsy-based diagnosis. A combination of these two methods provides even higher diagnostic accuracy, and LBC is considered an excellent method to evaluate these pathological samples.

## Introduction

The frequency of physical examinations and the introduction of computed tomography (CT) of the chest have increased the detection of peripheral nodular lesions in the lung (PNLL). These lesions used to be followed up without intervention or occasionally diagnosed by transbronchial aspiration biopsy/cytology (TBAB/C) and brush cytology. However, when the nodules are small and located in a peripheral region of the lung, tumor cells may not be collected due to the difficulty of direct aspiration. As a result, diagnostic accuracy has been far from satisfactory. An increasing number of institutes have carried out a CT-guided percutaneous lung biopsy (CTGLB) for PNLL (1,2). Furthermore, aspiration biopsy cytology (ABC) or cytology using lavage fluid from the aspiration needle have been used in these examinations. Using these methods, tumor cells can be collected from the samples for cytology, even in cases when a diagnosis is not reached by biopsy. However, as there are only a small number of cells in the aspiration needle and lavage fluid, it is imperative to succeed in collecting tumor cells for an accurate diagnosis. Conventionally, various methods have been used to smear the sample attached to the aspiration needle directly onto a glass slide, and to collect material by centrifugation of the lavage fluid (3). However, tumor cells are often desquamated from the glass slide during sample preparation. In addition, various contaminants, such as necrotic debris and inflammatory cells, are included in the sample and may interfere with the analysis. Several studies have attempted to solve these problems. The present study investigated liquid-based cytology (LBC), which has recently become a focus of attention.

LBC was first introduced in the area of gynecological cytology, and has since been developed. LBC enables not only reliable cell collection, but also the evaluation of uniform samples. In addition, certain useful features have been reported, including a reduction in problems in screening observations. Cytology using LBC has also been used in the pathological evaluation of other organs (4,5). Reports indicate that LBC is an excellent method for reliably collecting cells on glass slides from samples with a small number of cells (6–10). LBC has also been used recently for purposes other than cytological examination, for example, in molecular biological tests and in the genotyping of the human papilloma virus in patients with uterine cervical lesions (11,12). The fixation solution used in LBC is alcohol-based, so the destruction of the DNA and RNA is limited and the structure is stable for a relatively long period. Therefore, it has been used for various analyses in addition to cytology (13,14).

The present study investigated whether LBC, with these various advantages, is useful for the cytology of lavage fluid from the aspiration needle in CTGLB for PNLL.

## Materials and methods

### Case selection and specimen collection

Of the cases that underwent CTGLB at the Ibaraki Prefectural Central Hospital (Kasama, Japan) between 2004 and 2010, 130 were enrolled in this study. Biopsy samples were collected from these cases, and LBC samples were prepared from the lavage fluid of the aspiration needle. The cases were divided into two groups. One group was comprised of 73 cases in which tumor lesions were diagnosed or suspected from at least one of the following: Biopsy, LBC and the conventional method (CM); and in which partial lung resection was performed after thoracotomy or under thoracoscopy. CM is an ordinary sample processing process without the LBC method. These cases were diagnosed histologically from the resected specimens. The other group was comprised of 57 cases in which a definite diagnosis was not made clinically or by any other method. These cases were followed up radiologically and considered to have non-tumorous lesions, as the nodules showed no aggravation (nodule size increase), disappeared or remained unchanged. The study protocol was approved by the ethics committee of Ibaraki Prefectural Central Hospital. Written informed consent was obtained from the patients or patient’s family.

### Sample preparation

The biopsy tissue that was obtained was fixed immediately in 10% buffered formaldehyde solution, embedded in paraffin, sliced according to the CM and stained with hematoxylin-eosin for observation. The lavage fluid obtained by washing the aspiration needle following biopsy with physiological saline was centrifuged (1,700 × g for 10 min). The sediment was smeared on silane-coated slides using the wedge method and fixed immediately in 95% ethanol using the CM. LBC samples were prepared with ThinPrep2000™ (Cytyc Corporation, Boxbough, MA, USA), according to the manufacturer’s instructions. The centrifuged sediment was resuspended and fixed in cytopreservative solution, and smear samples were automatically prepared with ThinPrep, for which special filters and glass slides were set up. Samples constructed by the CM, and the LBC samples, were subjected to Papanicolaou staining for observation.

### Evaluation and classification

For diagnostic evaluation of the cytological samples prepared by LBC and the CM, two observers first observed and diagnosed them independently. When the diagnosis was inconsistent, the two observers reexamined the case under a double-headed microscope and reached a joint conclusion. A cytological diagnosis was made according to three grades: i) Benign, no malignant cells present; ii) suspicion of malignancy, presence of atypical cells suspicious of malignancy; and iii) malignant, presence of malignant cells. When malignancy was diagnosed, the histological types were estimated. The cytological, biopsy-based and radiological diagnoses were made in an independent and blinded manner, so that information from one method could not affect the diagnosis by other methods.

### Statistical analysis

The sensitivity, specificity and accuracy of these diagnostic methods were calculated. The χ^2^ test was employed for the comparison between two groups, and P<0.05 was considered to indicate a statistically significant difference.

## Results

### Sample preparation and evaluation

Samples were prepared and evaluated appropriately by LBC in 94/130 cases (72%) and by CM in 47/130 cases (36%). Inappropriate sample preparation, such as no cell collection on the glass slide, occurred by LBC in 36/130 cases (28%) and by CM in 83/130 cases (64%) (Table I). Table II shows the cases that could be evaluated according to the three-grade cytological criteria.

### Cytological findings

**Figure 1:**
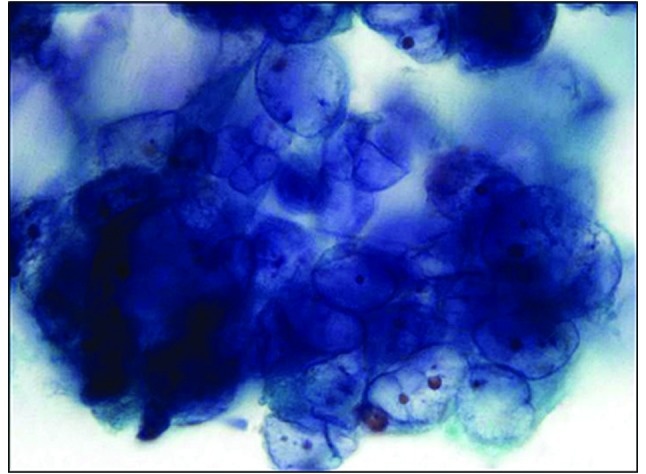
Cellular findings of lung adenocarcinoma prepared using the conventional method (CM). The nuclei of the tumor cells are swollen (Papanicolaou staining; original magnification, ×400).

**Figure 2:**
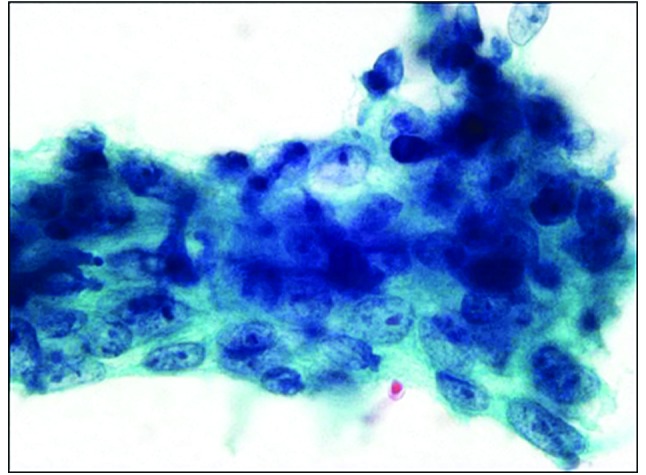
Cellular findings of lung adenocarcinoma prepared using the liquid-based cytology (LBC) method. The nuclei of the tumor cells are reduced in size and show fine chromatin and prominent nucleoli (Papanicolaou staining; original magnification, ×400).

**Figure 3:**
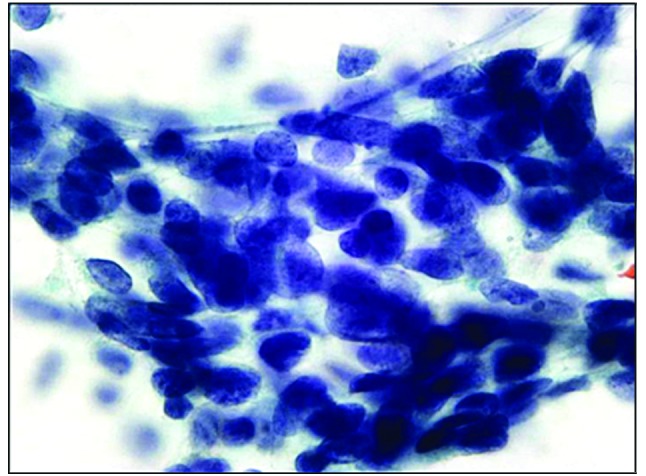
Cellular findings of lung small cell carcinoma prepared using the liquid-based cytology (LBC) method. Closely-knit syncytial clusters are observed with stripped and hyperchromatic nuclei (Papanicolaou staining; original magnification, ×400).

The cellular findings by LBC and the CM were as follows. In the squamous cell carcinoma cases, the cellular findings were comparable between the two methods. Contamination with necrotic debris and inflammatory cells in the background was limited, and the majority of tumor cells were scattered and present in an isolated or solitary manner. The cytoplasm of the tumor cells was dense and either eosinophilic or amphophilic. The nuclei were hyperchromatic with an uneven distribution of coarse granular chromatin, while the nuclear membrane was irregular and nucleoli were prominent. In the adenocarcinoma cases, cell clusters were three-dimensional or overlapping and maintained a sheet-like or papillary cluster. Small glandular lumina were also observed within the cluster. However, the tumor cells showed a slight difference in nuclear findings. Compared with LBC, swollen nuclei were prominent in the CM (Fig. 1). Conversely, in LBC, the nuclei were smaller than in the CM, and fine granular chromatin and prominent nucleoli were observed (Fig. 2). Small cell carcinoma cells exhibited a pavement-like arrangement with stripped and hyperchromatic nuclei (Fig. 3). In the metastatic tumor cases, tumor cells showed similar cellular findings to the primary lesion. In the cases of metastatic tumor derived from colon cancer, the clusters were composed of columnar tumor cells containing mucinous fluid or necrotic debris in the background.

### Histopathological correlation

The final histological diagnosis of the resected material, biopsy-based diagnosis and estimated histological diagnosis by LBC and the CM are shown in Table III. In the final diagnosis, there were 65 cases with primary tumors (64 malignant and 1 benign), 6 with metastatic tumors and 2 with non-tumorous lesions. Malignant tumors were diagnosed by biopsy in 64 cases. A total of 47 cases were diagnosed as malignant by LBC and 31 cases were diagnosed as malignant by CM. Malignancy was finally confirmed in the surgical specimens. Although the definite diagnosis was not obtained by biopsy-based examination, 10 cases were finally diagnosed as malignant in the resected specimens. Six of them were diagnosed as malignant or having suspected malignancy by LBC. In the remaining four cases, the samples showed no sign of malignancy or were inappropriate for evaluation by LBC. Furthermore, of the cases that obtained a definite diagnosis by biopsy, four had tuberculosis or inflammatory disease. Either their evaluation by LBC was benign or the samples were inappropriate.

### Statistical findings

LBC on its own provided a sensitivity of 68%, a specificity of 61% and an accuracy of 65%. A combination of biopsy and LBC provided a sensitivity of 94%, a specificity of 81% and an accuracy of 84% (Table IV).

## Discussion

With regard to the utility of ABC for PNLL, previous studies have shown that CTGLB cytology provides an accuracy of 67.6%. In the present study, inappropriate samples were eliminated by staining and the collected samples were evaluated immediately on site (3). This contributed to the high accuracy of this approach, which was superior to the conventional smear method, but inferior to LBC in the present study. The skill of the examiner and the diameter of the aspiration needle may have had a certain level of impact on the degree of accuracy. Furthermore, the procedure in which the aspiration needle touches the glass slide directly in the conventional smear method may have had a certain effect on the results. It is easy to obtain a large number of cells with this method, but it is not possible to examine the samples other than on a glass slide. In certain cases, this method is likely to cause mechanical damage to the biopsy material, which may occasionally affect the diagnosis.

LBC, initially introduced in the field of gynecology, has begun to be used frequently in other fields (15). Urine cytology has been employed most frequently at various institutes. Liquid samples are the subject of urine cytological examination, so there is the disadvantage that the cells may desquamate easily from the glass slide. Several methods have been devised to prevent this. Following reports that a large number of cells may be collected most efficiently by LBC, the diagnostic accuracy of urine cytology has improved (16,17). With regard to the application of fluid samples from the body cavity, diagnostic accuracy is not improved compared to the conventional CytoSpin method (18). In addition, LBC has been applied for lavage fluid of the brush in biliary disease (19), and the diagnostic accuracy was improved by combination with the CM. Moreover, LBC has been used for ABC for various diseases and organs other than those providing liquid samples. This application is aimed at avoiding the loss of cells and preparing samples efficiently, as ABC collects only a small number of cells (7–10).

To date, a few studies have used LBC for PNLL, similar to the present study. One study showed that, compared with the CM, the overall accuracy improved and the number of inappropriate samples decreased with LBC (20). In the present study, there was a large difference between LBC and the CM in terms of the sample number that could be evaluated. A large number of cells on the LBC slides improved the accuracy of the diagnosis. Conversely, more than half of the cases in the CM could not be evaluated, as insufficient numbers of cells were collected. The fact that there was a large difference in the percentage of samples that could be evaluated was attributable to several factors. Few cells were collected reliably and smeared on glass slides, and the cells were difficult to desquamate from the LBC samples. Although the diagnostic specificity of LBC was inferior to biopsy, LBC exceeded the CM in terms of sensitivity and accuracy. However, data from a study by Konofaos *et al* (20) differed greatly from the present study results, as it reported that the specificity was 100% for LBC and the CM. In the study, the tumors were resected by thoracotomy and all 80 were histologically diagnosed as benign or malignant and then analyzed. However, non-tumor cases were not included. In such groups, the sensitivities are calculated with the number of cases evaluated as positive by cytological diagnosis. However, it is difficult to understand how the specificities were calculated, as the non-tumor cases were not included. Moreover, a negative predictive value was also calculated (20). Wallace *et al* (21) constructed cell blocks from LBC samples and made a diagnosis. It was reported that the LBC samples were high in quality and that the construction of cell blocks enabled immunohistochemistry to be applied. Furthermore, although the analysis was made only for the case of small cell carcinoma of the lung, Kim and Owens (22) concluded that LBC should replace the CM in constructing pathological samples.

Endoscopic ultrasound-guided transbronchial or transesophageal lymph node aspiration (23) and aspiration biopsy under thoracoscopy (24) have been carried out in a number of institutes for lung hilar lymph nodes to determine the staging of tumor cases. Construction of LBC samples from these aspiration biopsy materials may be useful for various reasons.

In the present study, the number of cases diagnosed as malignant by LBC was comparable to that by biopsy. However, six cases were not diagnosed by biopsy, but were determined to be either malignant or suspected of malignancy by LBC. These results indicate that a combination of biopsy and LBC would increase diagnostic accuracy. In fact, sensitivity, specificity and accuracy all improved with this combination compared with either method on its own. Four cases that were diagnosed as being malignant in the resected specimens failed to be diagnosed as malignant by biopsy or LBC. In these cases, it was suspected that the tumors were not properly aspirated by biopsy or LBC.

There was no significant difference in the appearance pattern of tumor cells and the shapes of clusters between the two methods in the cases with adenocarcinoma that could be evaluated. While the nuclei of the tumor cells showed marginal swelling with the CM, this finding was either not observed or the nuclei tended to be smaller in size with LBC. These varying results may be attributable to the nuclei being swollen in physiological saline, but then reduced in size by alcoholic fixation in the LBC preparation solution.

LBC enables the evaluation of a large number of uniform samples and has several advantages in being able to collect material from samples containing only a few cells. Furthermore, liquid samples can be stored for a certain period of time prior to smearing and can be used for other analyses, including immunocytochemical analysis. In LBC samples, the cell membranes and the cytoplasmic and intranuclear antigenicity are maintained, and the samples are suitable for immunocytochemical analysis (25–27). In certain cases, immunocytochemical analysis using LBC samples would be useful for the determination of benign or malignant tumors in the lung (28). In addition, LBC has also been used for molecular diagnosis, such as in fluorescent *in situ* hybridization (29,30). Molecular targeting therapy has been carried out in inoperable cases with non-small cell lung carcinoma and in pre-operative neoadjuvant therapy. The indication for gefitinib therapy is currently evaluated based on the presence or absence of a mutation in the epidermal growth factor receptor gene. Biopsy samples and surgically-resected specimens have been used mostly for exploration, and cytology specimens may also be increasingly employed in the future (31,32). LBC is likely to become one of the applicable methods in this field.

In conclusion, CTGLB is likely to be employed more frequently in the pre-operative diagnosis of PNLL. Diagnostic accuracy is likely to improve as CT instrument and aspiration devices are developed. Furthermore, LBC is the most appropriate method to enable the collection of cells from samples containing only a few cells. Aspiration biopsy and LBC may be used in combination more frequently in the future, as this can improve the sensitivity, specificity and accuracy of a diagnosis.

## Figures and Tables

**Table I:** Suitability of the specimens for LBC and CM.

Suitability	LBC, n (%)	CM, n (%)
Adequate	94 (72)	47 (36)
Inadequate	36 (28)	83 (64)

LBC, liquid-based cytology; CM, conventional method.

**Table II:** Results of cytological evaluation by LBC and CM.

Evaluation	LBC, n (%)	CM, n (%)
Benign	47 (50)	32 (68)
Suspicious	24 (26)	9 (19)
Malignant	23 (24)	6 (13)

LBC, liquid-based cytology; CM, conventional method.

**Table III:** Details of histological subtype and the number of cases in the specimen from resection, biopsy, LBC and CM.

Histological subtype	Resection	Biopsy	LBC	CM
Primary tumor				
SCC	8	8	6	3
AC	46	37	33	24
SM	2	4	3	1
Other	8	0	2	1
Non-small	0	6	1	1
Malignant cells	0	0	2	1
AAH	0	1	0	0
Atypical cells	0	1	10	2
Benign tumor	1	2	0	0
Metastatic tumor	6	5	2	0
Non tumorous lesion	2	7	6	1
Inadequate	0	2	8	39
Total	73	73	73	73

SCC, squamous cell carcinoma; AC, adenocarcinoma; SM, small cell carcinoma; AAH, atypical adenomatous hyperplasia; LBC, liquid-based cytology; CM, conventional method.

**Table IV:** Reliability of biopsy, LBC and the two combined.

Reliability	Biopsy	LBC	Biopsy + LBC
Sensitivity	86	68	94
Specificity	89	61	81
Accuracy	87	65	84

All values are percentages; LBC, liquid-based cytology.
